# Task-shifting: experiences and opinions of health workers in Mozambique and Zambia

**DOI:** 10.1186/1478-4491-10-34

**Published:** 2012-09-17

**Authors:** Paulo Ferrinho, Mohsin Sidat, Fastone Goma, Gilles Dussault

**Affiliations:** 1 International Public Health & Biostatistics Unit and CMDT, WHO Collaborating Centre for Health Workforce Policy and Planning, Instituto de Higiene e Medicina Tropical, Universidade Nova de Lisboa, Lisbon, Portugal; 2 Faculty of Medicine, Universidade Eduardo Mondlane, Maputo, Moçambique; 3 Faculty of Medicine, University of Lusaka, Lusaka, Zambia

## Abstract

**Background:**

This paper describes the task-shifting taking place in health centres and district hospitals in Mozambique and Zambia. The objectives of this study were to identify the perceived causes and factors facilitating or impeding task-shifting, and to determine both the positive and negative consequences of task-shifting for the service users, for the services and for health workers.

**Methods:**

Data collection involved individual and group interviews and focus group discussions with health workers from the civil service.

**Results:**

In both the Republic of Mozambique and the Republic of Zambia, health workers have to practice beyond the traditional scope of their professional practice to cope with their daily tasks. They do so to ensure that their patients receive the level of care that they, the health workers, deem due to them, even in the absence of written instructions.

The “out of professional scope” activities consume a significant amount of working time. On occasions, health workers are given on-the-job training to assume new roles, but job titles and rewards do not change, and career progression is unheard of. Ancillary staff and nurses are the two cadres assuming a greater diversity of functions as a result of improvised task-shifting.

**Conclusions:**

Our observations show that the consequences of staff deficits and poor conditions of work include heavier workloads for those on duty, the closure of some services, the inability to release staff for continuing education, loss of quality, conflicts with patients, risks for patients, unsatisfied staff (with the exception of ancillary staff) and hazards for health workers and managers. Task-shifting is openly acknowledged and widespread, informal and carries risks for patients, staff and management.

## Background

In many African countries, basic level workers, and even untrained auxiliary staff and community health workers, assume roles and perform activities legally reserved for mid- or high-level cadres of workers [1]. This is informally acknowledged, but rarely documented, and it is generally ignored in human resources for health (HRH) planning.

We describe the task-shifting between different cadres in health centres (HC) and in district level hospital settings in the Republic of Mozambique and the Republic of Zambia, and present the perceptions of health workers of their skills in relation to their actual tasks.

This type of information is important for policy and planning purposes because it serves to verify the existence of informal task-shifting, allowing a clearer idea of the baseline situation to be changed through scaling-up efforts. Also, the perceptions of health workers of the various dimensions of their work are a determinant of their willingness to change their scope of work and responsibilities. To know the opinions of health workers is important because they are active agents, faced with many competing incentives and constraints, in dynamic labour markets [2].

The first section of the paper is a brief description of the health workforce situation in Mozambique and Zambia, two countries which were selected for their recent health workforce planning efforts in which the authors participated [3,4]. The methods used to collect and analyse data are presented followed by the results and their discussion.

### Country contexts

Mozambique and Zambia are countries covering large areas, with estimated populations of 22 and 13 million, respectively. They have similar levels of urbanization, health and HRH indicators (Table 1). There are important political and cultural differences: Mozambique is a former Portuguese colony and is recovering from a civil war that followed an armed struggle for independence. Zambia, a former British colony, gained independence peacefully and never experienced civil war.

**Table 1:** Country profile: Mozambique and Zambia (2009–2010)

		**Mozambique**	**Zambia**
Area (km^2^)		801 590	752 618
Population		22 million (2008)	13 million (2008)
Urbanisation rate		37%	35%
Gross national product/purchasing power parity per capita (int $)		770	1230
Population living on < $1/day		74.7%	64.3%
Human Development Index (2010) (both countries are below the Sub-Saharan Africa average)		0.317,	0.425,
Adult literacy (2000–2007)		44%	71%
Health indicators	Life expectancy at birth (years)	42.1	42.4
	Maternal mortality/100 000 live births	520	830
	Infant mortality/1000 live births	90	92
	Malaria mortality/100 000 population	92	121
	Tuberculosis in people positive for HIV/100 000 population	36	18
	Prevalence of HIV in people 15–49 years of age	12.5%	15.2%
Human resources for health situation	Assisted deliveries	48%	47%
	Physicians/10 000 population	<0.5	1
	Nurses and midwives/10 000 population	3	7
	Pharmaceutical personnel/10 000 population	<0.5	<0.5
	Dental personnel/10 000 population	<0.5	<0.5

HIV: human immunodeficiency virus.

Source: WHO. World Health Statistics 2010 and hdrstats.undp.org/en/countries/profiles/.

Zambia’s health policy was formulated in the *National Health Strategic Plan* (NHSP 2006–2010) [5]. The Ministry of Health estimates that its health workforce corresponds to less than half of the requirements to deliver basic services [5,6]. HRH issues are identified as a priority, which the *Human Resources for Health Strategic Plan 2006–2010* addressed [6].

In Mozambique, health policy directions are formulated in the *Plano Estratégico do Sector Saúde* (Health Sector Strategic Plan [7]), which identifies the deficit of skilled health workers as a major challenge for the implementation of the health policy. In response, the government has adopted a plan for human resources development (*Plano de Desenvolvimento de Recursos Humanos*) for 2008–2015 [8].

In both countries, in addition to limited funding [4], the greatest obstacle to increasing the workforce is the insufficient capacity for training new workers. This is why task-shifting is regarded as a realistic, efficient and rapid way to scale-up access to qualified services, at a much lower cost than training more physicians and nurses [9].

Two sets of questions triggered our research. First, does task- shifting exist, and if so, to what extent and in what form? Second, how great is health workers’ willingness to perform expanded functions and how do they assess their competencies to do so? What are the views of managers of health services on task-shifting?

## Methods

We conducted individual and group interviews with key health personnel and focus group discussions with health workers from the civil service (Additional file 1: Annex I – downloadable file), with a view to gather information of experiences of task-shifting and on perceptions of workers and managers on their competencies and on their working conditions in general.

We first selected HC and district hospitals (DHs) to ensure a representation of urban and rural facilities and of all levels of services: we included at least one facility from each area of great isolation. This selection was done with the support and approval of Ministry of Health officials. All facilities contacted by telephone agreed to participate. The managers of each facility assumed the responsibility to mobilize their staff (we do not have information on staff refusal to participate).

On average, two focus groups were conducted by each facility: one with technical staff and one with ancillary staff. Where the number of technical staff allowed it, we organized focus groups for a specific cadre (e.g. nurses) (Additional file 2: Annex II – downloadable file). In the case of managers, individual interviews or interviews with two respondents were conducted.

Interviews (Additional file 3: downloadable file for interview schedule) and discussions shared the same set of issues for discussion and were recorded with the consent of the participants, transcribed and subjected to open, qualitative content analysis [10].

## Results

### Experiences and opinions of health facilities’ staff and managers

In the following sections, we describe experiences of task-shifting and present opinions expressed by managers, technical staff and non-professional ancillary staff working in hospitals and HCs.

### Experiences of task-shifting

#### Health facility managers

In both countries, health facility managers experience a significant staff deficit at all levels of care. But, in Mozambique it is felt that the deficit situation is improving, particularly in the urban areas, therefore less use is made of non-professional ancillary staff for clinical activities. In district health facilities, staff deficits are reported:

*… in Mitande* [Niassa] *there is only one nurse… he does dental chores … conducts labours … consults with patients besides doing his own nursing work … He runs the dispensary, …*

We have some health posts where the only staff present is auxiliary midwives and a maid. In the absence of the auxiliary midwife the maid takes over … even antenatal care …

Task-shifting is described by managers as very frequent in the health facilities of both countries*:*

*… [we cope] because people are doing more than their job description … like the maids … the maid may help the nurse at the end of the day. Even helping to take bodies to the mortuary…* (Lusaka HC)

Making use of underskilled or unskilled personnel to ensure the running of a health facility is part of the difficulty in improving the quality of the services provided:

*There are situations where other people, mostly helpers, do nurses’ … They may help with preparing bodies for the mortuary, bed making, registration in OPD and cleaning instruments in theatre … when doctors are not there [Clinical Officers are asked to assist] with the ward rounds… There are even situations when patients are clerked by nurses because there were no clinical officers … Nurses may also have to do other doctors’ jobs, like withdrawing of blood* (Kafue DH)

*In labour room … maids are mobilized to assist [skilled nurses]…* (Niassa Regional Hospital (RH)

*… besides, they do not value confidentiality …* (Gaza RH)

Managers gave other examples of task-shifting:

*For example … because we lack trained counsellors, antiretroviral counselling is conducted by a dental technician and a physiotherapy technician … Another example is that of the radiology technician who is also head of the financial department, for lack of someone else* (Nampula RH)

*… we have one nurse who helps in the radiology department and another in … in charge of pharmacy … the nurse working at the pharmacy, she is there the whole time…but other nurses come to help out at the pharmacy as well … in the X-ray department … may be 20% or 25% of the time* (Mpanshyia Hospital)

In situations of chronic deficit of personnel, nurses “*will train their ancillary staff to assume clinical chores*” (Nampula HC).

Managers acknowledge that this task-shifting is usually unplanned, but when it is, it is never done in writing: “*… the allocation of new tasks to the health workers is made verbally …*” (Nampula HC).

#### Care providers

The experiences of care providers coincide, to a large extent, with those of managers.

Except for Maputo HCs, where the situation was considered acceptable, all technical staff interviewed complained of lack of staff and of being compelled to assume a diversity of extra tasks.

*As enrolled nurses, we are not supposed to be delivering (in the labour room, but we do it). We are also doing [patient] screening [in OPD] … MCH activities [which] are supposed to be done by a qualified person … suturing and doing cannulations … minor surgery – I&D [incision and drainage]… giving IV drugs…we even do death certification [on behalf of the clinical officer]. We also do the counselling and testing for HIV… and also testing for Hb, RPR and RDT [rapid diagnostic tests] for malaria … administrative work … we don’t even know our job description These other extra duties take most of our time”* (Zambian Rural HC)

*… the cleaners … do chores such as damp dusting, bed making, weighing babies and mothers in MCH, taking temperatures and other vitals, etc.… with no training other than … on-the-job. They learnt by doing alongside the nurses…[but even with their help we, nurses] are not enough* (Lusaka HC)

A number of nurses and of other health personnel say that their initial training prepared them to some of the task-shifting they assume:

*… when we are at nursing school, we get exposure to some of these areas like we go for clinics, we also work in the wards, so that whatever small experience we have, when we come in the field, we are not surprised, we find the situation as bad as we were told, we just fit in* (Zambian Rural HC),

*We, general nurses, learn everything … if you take me now and place me in maternity I will be prepared to work there. I can screen. I can handle some small surgery. I can work in the wards. We are really taught a bit of everything [during our initial training]* (CS Maputo)

This does not seem to apply to some midwives:

*I am a midwife, after losing the general nurse [at the HC] I had to assume tasks for which I felt I was not prepared. Therefore I referred a lot …*(Niassa HC)

In addition to lack of preparation, the absence of adequate equipment causes another major bottleneck:

*There are things that the dental therapist wants to do but cannot do them because the equipment is not adequate …*(Lusaka HC).

In fact, this releases staff from the duties they were trained for to assume other tasks that are shifted to them.

Some of the task-shifting observed takes place because of the lack of transport to refer patients:

*… when there are difficulties with transport to take the patient to the referral hospital we are forced to take on some very complex tasks …* (Nampula HC)

Technical staff shows openness to scaling-up the competencies of untrained personnel and relatives of patients, as a way to increase the capacity to cope with heavy workloads:

*… in the wards the relatives there really assist us in taking care of the patient* (Mpanshyia RH)

*In Maternal and Child Health, we have community health workers … who help weigh babies at the under-5 clinic and … mothers at the ante-natal clinic* (Mpanshyia RH)

#### General non-professional ancillary staff

These staff members clean, do the laundry and cook. In day-to-day life, on a need to do basis, they assist with a number of tasks, for which they have no training.

*I am an indoors cleaner. I help the nurses do most of their work. I do bed-making, bring bed-pans to the patients, monitor temperatures, and also wait on patients. I help cooking for the patients, and sometimes I go to help in the lab to do finger pricks for the slides, and do tests such as RPR, pregnancy tests. I also help with growth monitoring. I also look after the stores for World Food Programme “food-for-work” project. I keep records of the food in the stores and do stores controlling. Sometimes you just arrive, before you start cleaning up, you have to make beds, go to collect linen, etc. … These take three quarters of my time* (Zambian rural HC)

*When I was in the district, my nurse had to go away for one month and was not replaced. I was all by myself … I had to do dressings, suture wounds, drip patients, assist women in labour, and still do my own cleaning job* (Gaza HC)

*I was not trained. But as I saw so many being done I did it myself. I thought it was normal* (Niassa HC)

At Maputo General Hospital (GH), Mozambique, a different situation is observed. Only occasionally do ancillary staff members support the midwives, handing over the equipment for example, but they never conduct any clinical work on their own. Most received some training before starting their work in the hospital. They are happy with their situation. This contrasts with what is observed in all other hospital facilities visited, where they assume all sorts of tasks including clinical ones,

*In registry, there is also work which is not supposed to be done by us, but you find yourself maybe tallying diagnosis or other treatment [of data]… when a patient comes … unable to walk or talk, you are forced to get the patient to OPD … there you find there is no nurse, maybe she’s somewhere else, you will be forced to do the nurse’s job, for the sake of assisting that patient because you can’t just leave him collapse* (Kafue RH)

*I helped in maternity. I conducted deliveries. I did that to help the nurse. Let’s suppose that five or six mothers come all in labour. Then the nurse would ask for my help. I had to help. I could not let the mothers push out the baby on the floor. Of course, I would put on my gloves and would run to help the nurse* (Nampula RH)

### Perceptions and expectations regarding task-shifting

One district health director in Niassa, Mozambique, considers that, compared with other provinces, the level of staffing in his district can be considered reasonable *(“in the district head office we have enough staff to respond to the flow of work …”)*, but bad management of the continuing education of his staff by the provincial head office creates disruptions:

… the staff who remains in the health centre may be overloaded by work … continuing education initiatives occur spontaneously, unplanned, creating service constraints …

…[In] Mitande … the situation gets really serious when [the single nurse on duty] is called for continuing education …

The most important deficit observed is for maternal and child health nurses and auxiliary clinical officers (agentes de medicina). The director considers that the major obstacle in placing staff in isolated rural areas is related to the lack of accommodation; this aggravates the staff deficit that leads to task-shifting.

HC managers are usually health professionals (most frequently nurses) who cumulate management and clinical tasks, spending most of their time on the latter, thus neglecting managerial activities, for which they are not prepared:

*… the HC should have an administrator … some of the administrative activities are difficult … experience is not enough to carry them out adequately … human resources management has been most difficult …* (Niassa HC)

Hospital managers cumulate management functions with district management and clinical duties:

*We have to care for patients in the wards and to see them as outpatients. In practice this means that administrative work is done in late afternoons, except in days when I have to participate in the activities of District Government … which pushes my ward visiting times to after 6 pm* (Gaza RH).

Management and administration are seen as a burden for which hospital managers feel ill prepared:

*I was frustrated at the beginning, because my training was as a MCH nurse and not in administration … with time I got used … I spend 65% of my time with administration and 35% with nursing duties … I am not happy with this situation. I would rather spend all my time with clinical chores … I have difficulty with statistical reports and I should have a computer to help me with my work* (Maputo GH)

This overload could be reduced if there were more and better trained administrative personnel. In their absence, highly trained staff members have to spend their time doing general basic chores:

*We should have more administrative staff … There are chores that do not need to be done by myself but I have to get involved because the staff available is not skilled enough…* (Niassa RH)

*“The main skills lacking in the hospital are nursing and midwifery. These are compensated by the use of part time nurses and by nurses in the establishment who do calls … but even so they are not enough to cover all the night shifts* (Kafue DH)

According to the managers, the situation of deficit prevents proper in-service training:

*… the problem is that we are so short of staff that even training is difficult, in-service training which was supposed to be routine is lacking because there is no time to remove staff from their posts, but we know that, that is the only way to overcome the quality crisis …* (Mpanshyia Hospital)

Managers report absenteeism for illness as a problem:

*…because of staff members that are frequently ill, we do have occasional shortages of non-professional ancillary staff and of other health workers* (Maputo HC)

They consider that, in general, the situation is more difficult in rural than in urban health centres: *“…we are supposed to have three doctors but we only have one…”* (Lusaka HC).

Managers acknowledged that informal task-shifting involves risks for them:

*…when we had maternal deaths, it wasn’t only the nurses who were asked to write a report, even the sister in-charge, even the nurses who were there when the mother died, they also wrote the report. So management is also liable [for the hazards resulting from task-shifting]* (Lusaka HC)

Most who work overtime or assume new functions are not rewarded financially as this is not allowed by the rules of the Ministries of Health:

*… maybe the lack of financial rewards is not the worst of it. Also, there is a lack of recognition of the add-on work that they assume…* (Maputo GH)

Managers consider that, overall, staff members are unhappy, *“We notice that workers are not happy…”* (Maputo GH).

As with their experience of task-shifting, the perceptions and expectations of care providers coincide, to a large extent, with those of managers.

Again, excepting for Maputo HCs, where the situation was considered acceptable, technical staff feel compelled to assume a diversity of extra tasks out of comradeship—or on instruction from management, *“… the Provincial Health Director just told me to do it, to assume the responsibility for the expanded programme of immunization”* (Niassa HC), and, *“Orders are orders …”* (Nampula HC).

Care providers consider there to be several consequences of staff deficit and the poor conditions of work: a heavier workload on the staff on duty; the closure of services, and its consequent reduction of access; the inability to release staff for continuing education; poorer quality and poorer efficiency as qualified staff has to perform menial tasks; conflicts with patients; and increased risks for health workers and users of services. Hospital technical staff considers that the staffing shortage in hospitals is critical and affects skilled and unskilled staff categories, resulting in significant shifts of tasks among different categories of health workers,

*“We have five staff members involved with patient screening. We see so many patients, too many patients. I believe that each day each one of us sees up to 75 or even 85 patients.****That is too much****…”* (Maputo HC),

*“There are not enough staff members. Therefore we are****forced to close some sectors****, because of this lack of staff …”* (Maputo HC),

*“… so there is a problem,****we are not being looked after properly****[as far as continuing education is concerned] when there is any new program on board …”* (Lusaka HC),

*“if you are overworked, your****care will lack quality****”* (Zambian rural HC),

*“… sometimes I have to interrupt what I should be doing [consulting with patients] to assist with dressings, injections …****Patients get very angry****when they have to wait for a long time until I resume consulting them …”* (Nampula HC),

*“… you are alone in labour ward… and there are 10 women about to deliver, so the risk of infecting each other is high. People would rather put on four pairs of gloves and just keep on removing the top ones. And for example you find 2 or 3 mothers are pushing, then the babies are there on the vulva, which one do you get first? If you get that one, maybe****that other one will drop on the floor, again you will be the one to answer for that****”* (Lusaka HC),

*“At the X-rays … we are supposed to have dosage monitoring badges, so we have requested from the district and the regulation protection board … we have been working for almost a year but****we don’t have those badges****”* (Lusaka HC)

*“because of shortage of staff … our dental officer is in charge of tuberculosis and leprosy control,****even if he is not competent to run those programmes****…”* (Nampula RH)

*“… when I come in the morning I find there are few cleaners at the hospital and they are held up somewhere, in that case I just get the broom and clean my department and then I start working because I won’t wait for that cleaner to come and clean for me to start,****I have patients waiting****. So, I was trained as a physiotherapist but I sweep …”* (Kafue RH).

*“I am a clinical officer for anaesthesia … in the theatre there are the three of us, so when the procedure is going on, everything that is needed I have to do it.****I am a circulating nurse, I am a runner, a porter****…”* (Kafue RH),

Suggested solutions include:

… *training the ancillary staff in routine duties such as feeding patients, taking BP, temperature and other vitals, so as to be more effective …* (Zambian rural HC);

*Continuing education programmes have to be decentralized to the periphery* (Niassa RH);

*[as the] auxiliary nurse carries almost the same tasks as a mid-level nurse … it would be right if that auxiliary nurse could be promoted to mid-level nurse by a distance learning course* (Nampula RH).

## Discussion

Our results are consistent with other studies from Mozambique and Zambia in terms of the extent of task-shifting and sharing we have observed [11,12]. Our studies further specify the type of task-shifting and document the opinions of staff about the situation.

In Mozambique, a recent study on nursing has shown that auxiliary nurses tend to assume nursing responsibilities above their level of training, particularly in isolated settings of lesser complexity. There was a significant overlap of tasks between nurses (all levels) and clinical officers and auxiliary clinical officers [12]. This is consistent with our findings. This suggests that existing auxiliary level staff could be upgraded to mid-level professional skills. Another consideration is that the training of new auxiliaries should probably be abandoned in favour of mid-level workers.

The delegation of tasks from one health care provider to another, advocated in the 2006 *World Health Report*[13]*,* has been used in many countries on a routine basis for decades, especially in understaffed rural facilities, to enhance access and to reduce costs [14]. One of the earliest and most systematic studies was undertaken in the 1970s and 1980s in the Democratic Republic of the Congo (then Zaire) [15].

It was recognized, from the time of Mozambican [16,17] and Zambian independence [18], and even before [19-21], that there was a need to develop health services with less skilled trained cadres, such as auxiliary health workers or even traditional personnel such as traditional birth attendants [22,23]. This justification was that many activities of doctors and nurses could be carried out at a lower cost by persons with shorter training, which allows for faster entry into the labour market and is significantly cheaper.

This recognition did not come easily, nor was it always the result of systematic planning, and not all stakeholders supported it. In most instances, task-shifting occurs in the absence of adequate support, such as access to means of communication, supervision and mechanisms of referral. It also often takes place in an environment in which basic amenities—housing, water, electricity and sanitation—are lacking. Even so, some cadres, like clinical officers in Zambia and surgical technologists in Mozambique [24], are examples of effective formal processes of task-shifting.

Another form of task-shifting is observed when workers are trained beyond their legal scope of practice, as a strategy to facilitate the implementation of specific programs. For example, in Zambia this is done for nurses and environmental health technologists: during their pre-service training they are taught to screen patients and to prescribe for the most common conditions. Also in Zambia, an Integrated Competence-based Training programme has been developed for those already working in the system. It gives front-line health cadres (clinical officers, nurses, environmental health technologists) basic competencies in the delivery of the entire health care package at subdistrict level. These initiatives prepare Zambian health workers to substitute other cadres when the need arises [5,6]. In Mozambique, all categories of health workers receive training in minor surgical procedures, injections, and management of a febrile patient etc. The training of clinical officers as surgical technologists (técnicos de cirurgia) is another example from Mozambique [24].

## Conclusions

The consequences of the staff deficit and the poor conditions of work include a heavier workloads for the staff on duty, the closure of some services reducing patient access, the inability to release staff for continuing education, poorer quality, conflicts with patients, risks for patients, unhappy staff (with the exception of ancillary staff) and hazards for health workers and managers.

Task-shifting alone cannot respond to the needs of poor countries, especially if other issues are not addressed at the same time: inadequate facilities, lack of or faulty equipment, lack of transport for field work and for patient evacuation, unreliable electrical power supply, inadequate refrigeration facilities, lack of accommodation for staff, and inadequate continuing education efforts, among others.

Solutions cannot ignore the extent of the crisis, the maldistribution of resources [25,26], the insufficient training capacity, the imbalance of skills and the ancillary staff “reserve for rapid expansion of the skills base” within the public sector facilities. This paper shows that workers have clear ideas about how to improve the availability of services; it remains to be seen if decision-makers will listen to them.

## Competing interests

The authors declare no competing interests.

## Authors’ contributions

PF, MS and FG participated in the design of the study, in the data collection and analysis, and carried out the drafting of the manuscript. GD participated in the manuscript drafting. All authors read and approved the final manuscript.

## Supplementary Material

Additional file 1 Annex I Data collection sites. Click here for file

Additional file 2 Annex II Characterization of study participants. Click here for file

Additional file 3 Interview/focus group schedule of topics for discussion. Click here for file
