# Supplementary material for: Task-shifting: experiences and opinions of health workers in Mozambique and Zambia
Source: Hum Resour Health. 2012 Sep 17;10:34. doi: 10.1186/1478-4491-10-34 (PMC3515799; doi:10.1186/1478-4491-10-34)
Supplement: Additional file 1 — Annex I Data collection sites. [file 1478-4491-10-34-S1.docx]

Annex I Data collection sites

| **Province/Region** | **District/Health Area** | **Health facility** |
| --- | --- | --- |
| MOZAMBIQUE | | |
| Nampula (all rural) | Muecate | Muecate HC |
|  | Anchilo | Anchilo HC |
|  | Nacavala | Nacavala HC |
|  | Monapo | Monapo HC |
| Niassa | Cuamba (rural) | Cuamba HC |
|  | Mandimba (rural) | Mandimba District office |
|  | Lichinga (urban) | Chivaule HC |
| Gaza | Xai-Xai (urban) | Chicumbane RH |
|  |  | Chipenhe HC |
|  | Bilene (rural) | CS Joaquim Chissano |
| Maputo City (all urban) | Bairro de Chamanculo | Chamanculo GH |
|  | Bairro de Zimpeto | Zimpeto HC |
|  | Bairro de Bagamoio | Bagamoio HC |
|  | Bairro 1 de Junho | 1 de Junho HC |
| ZAMBIA | | |
| Lusaka | Chongwe (C district) (rural) | Level 1 Mpanshyia hospital |
|  | Kafue (B district) (rural) | Level 1 Kafue hospital |
|  | Lusaka (A district) (urban) | Chelstone public Urban HC |
|  | Luangwa (D district)(rural) | Luangwa Boma Rural HC |

GH, general hospital; HC, health centre; RH, regional hospital. The Zambian level 1 hospitals are equivalent to the Mozambican Rural and General (urban) Hospitals: all are considered district hospitals. The districts in Zambia are categorized from A to D with districts under D being the most disadvantaged or extremely rural; most districts in Western Province, North-Western Province, Luapula Province and half of the districts in Northern Province are classified as extremely rural; a retention scheme is being implemented for rural and extremely rural districts (categories C and D).
