# Supplementary material for: Task-shifting: experiences and opinions of health workers in Mozambique and Zambia
Source: Hum Resour Health. 2012 Sep 17;10:34. doi: 10.1186/1478-4491-10-34 (PMC3515799; doi:10.1186/1478-4491-10-34)
Supplement: Additional file 2 — Annex II Characterization of study participants. [file 1478-4491-10-34-S2.docx]

Annex II Characterization of study participants

| **Province/** **Region** | **District/Health Area** | **Health facility** | **Characteristics of study participants** |
| --- | --- | --- | --- |
| MOZAMBIQUE | | | |
| Nampula | Muecate | Muecate HC | Group interviews with managers: one medical technician (M), one mid-level nurse (M), one administration technician (M) |
|  |  |  | Focus group with technical staff: three auxiliary nurses (one F), one MCH mid-level nurse (F), three auxiliary midwives (F), one mid-level nurse (F) |
|  |  |  | Focus group with non-professional ancillary staff: four cleaners (one F) |
|  | Anchilo | Anchilo HC | Focus group with managers: one medical technician (M), one hospital administration agent (M), one mid-level nurse (M) |
|  |  |  | Focus group with technical staff: one agent of medicine (M), two MCH assistant nurses (F), two auxiliary nurses (F), one auxiliary midwife (F), one medical technician (M), one preventive medicine agent (M), one dentistry agent (M), one pharmacy agent (M) |
|  |  |  | Focus group with non-professional ancillary staff: six cleaners (three F), one driver (M) |
|  | Nacavala | Nacavala HC | Focus group with all staff: manager - one medicine agent (M); technical – one preventive medicine agent (F), one MCH assistant nurse (F); ancillary – two cleaners (F) |
|  | Monapo | Monapo RH | Focus group with managers: one doctor (F), one mid-level nurse (M), one accounting technician (F) |
|  |  |  | Focus group with technical staff: two mid-level nurses (one F), three assistant nurses (two F) |
|  |  |  | Individual interviews with technical staff: one BA level surgical technician (M), one medical technician (M) |
|  |  |  | Focus group with non-professional ancillary staff: five cleaners (three F) |
|  |  | Monapo HC | Individual interview with manager: one assistant nurse (M) |
|  |  |  | Focus group with technical staff: one MCH assistant nurse (F), one assistant nurse (F), one auxiliary nurse (M) |
|  |  |  | Focus group with non-professional ancillary staff: four cleaners (three females and one male ) |
| Niassa | Cuamba | Cuamba RH | Focus group with managers: three doctors (M), one public administration technician (M) |
|  |  |  | Focus group with technical staff: two mid-level nurses (one F), one laboratory technician (one M), one rehabilitation technician (M), one assistant pharmacist (M), one theatre technician (M), two MCH assistant nurses (F), one anaesthesia technician (M) |
|  |  |  | Focus group with non-professional ancillary staff: seven cleaners (three F) |
|  |  | Cuamba HC | Individual interviews with managers: one medical technician (F), one assistant administration technician (F) |
|  |  |  | Focus group with technical staff: one preventive medicine technician (M), one agent of medicine (M), one MCH assistant nurse (F) one auxiliary nurse (F) |
|  |  |  | Focus group with non-professional ancillary staff |
|  | Mandimba | Mandimba District office | Individual interview with manager: one medical technician (M) |
|  | Lichinga (urban) | Chivaula HC | Individual interview with manager: one assistant MCH nurse (F) |
|  |  |  | Focus group with technical staff: one auxiliary midwife (F) |
| Gaza | Xai-Xai (urban) | Chicumbane RH | Group interview with managers: one general medicine doctor (F), one medical technician (M) |
|  |  |  | Focus group with technical staff: two assistant nurses (one F), two MCH assistant nurses (F), two agents of medicine (F) |
|  |  |  | Focus group with non-professional ancillary staff: five cleaners (F) |
|  |  | Chipenhe HC | Individual interviews with one MCH assistant nurse (F) |
|  | Bilene | CS Joaquim Chissano | Individual interviews with one MCH assistant nurse (F), one pharmacy assistant (F) |
| Maputo City (all urban) | Bairro de Chamanculo | Chamanculo GH | Group with managers: one public administration technician (M), one hospital administration technician (M) |
|  |  |  | Focus group with technical staff: two MCH mid-level nurses (F), one MCH assistant nurse (F), one mid-level nurse (administration) (F), one preventive medicine technician (F) |
|  |  |  | Focus group with non-professional ancillary staff: three cleaners (two F) |
|  | Bairro de Zimpeto | Zimpeto HC | Focus with managers: one MCH mid-level nurse (F), one MCH auxiliary nurse (F), one hospital administration agent (M), one high school graduate (F) |
|  |  |  | Focus group with technical staff: one mid-level nurse (M), one auxiliary nurse (F), one agent of medicine (M), one medical technician (F) |
|  |  |  | Focus group with non-professional ancillary staff: three cleaners (two F) |
|  | Bairro de Bagamoio | Bagamoio HC | Focus group with technical staff: three MCH assistant nurses (F), one MCH mid-level nurse (F), one laboratory technician (M), one agent of medicine (F) |
|  |  |  | Focus group with non-professional ancillary staff: five cleaners (four F) |
|  | Bairro 1 de Junho | 1 de Junho HC | Individual interview with one assistant administrative technician (F) |
|  |  |  | Focus group with technical staff: one medical technician (F), two agents of medicine (one M), one mid-level nurse (F), one assistant nurse (F) |
|  |  |  | Focus group with non-professional ancillary staff: six cleaners (five F) |
| ZAMBIA | | | |
| Lusaka | Chongwe | Level 1 Mpanshyia hospital (rural) | Group interview with management: one registered theatre nurse, one registered nurse, one doctor, one clinical officer (sex not registered) |
|  |  |  | Focus group with technical staff: three enrolled nurses (one F), one enrolled midwife (F), one higher degree nurse (F), one registered nurse (F) |
|  |  |  | Focus group with technical staff: one physiotherapy technologist (F), one environmental health technologist (M), one laboratory technician (F) |
|  |  |  | Focus group with non-professional ancillary staff: two cooks, one driver, one mortuary attendant, one registry clerk, one laundryman (sex not registered) |
|  | Kafue | Level 1 Kafue hospital (rural) | Group interview with management: one doctor (M), one matron (F) |
|  |  |  | Focus group with technical staff: one pharmacy dispenser (F), two clinical officers (F), one physiotherapy technologist (F), one medical licenciate (F), one clinical officer (anaesthesia) (F), one radiographer (M), one nutritionist (F) |
|  |  |  | Focus group with non-professional ancillary staff: 21 cleaners (sex not registered) |
|  | Lusaka | Chelstone public Urban HC | Group interview with management: one clinical officer (M), one registered midwife (F), one registered nurse (F) |
|  |  |  | Focus group with technical staff: five enrolled nurses (F), one enrolled midwife (F), one clinical officer (M), one dental therapist (M) |
|  |  |  | Focus group with technical staff: one laboratory technician (M), one physiotherapy technologist (F), one pharmacy dispenser (F), one radiographer (M) |
|  |  |  | Focus group with non-professional ancillary staff: 12 cleaners (sex not registered) |
|  | Luangwa | Luangwa Boma Rural HC | Focus group with technical staff: four enrolled nurses (four F), one pharmacy dispenser (F), one registered nurse (F) |
|  |  |  | Focus group with non-professional ancillary staff: two cleaners (one F), one cashier (F), one watchman (M) |

F, female; GH, general hospital HC, health centre; M, male; MCH: maternal and child health ; RH, rural hospital. The Zambian level 1 hospitals are equivalent to the Mozambican Rural and General (urban) Hospitals: all are considered district hospitals.
