# Supplementary material for: Task-shifting: experiences and opinions of health workers in Mozambique and Zambia
Source: Hum Resour Health. 2012 Sep 17;10:34. doi: 10.1186/1478-4491-10-34 (PMC3515799; doi:10.1186/1478-4491-10-34)
Supplement: Additional file 3 — Interview/focus group schedule of topics for discussion. [file 1478-4491-10-34-S3.docx]

**Interview/focus group schedule of topics for discussion**

| 1. *Opening question*: If you think about what you were trained for, your post and what your job description expects you to do, and what you actually do on your day-to-day activities, what are the aspects of what you do that are not in harmony with your training and/or your post and/or job description? • *Clarify that everyone knows about their job description. If not, explain that it is what they were contracted to do.* 2. Clarify if functions and activities not in harmony with training and/or post or job description are occasional or are actually a major component of the job. • *Try to understand how major (quarter of the day, half the day …)?* 3. Clarify what the nature of those functions is? • *Clinical? Domestic? Administrative? Clerical? Other?* 4. Clarify if functions and activities not in harmony with training and job description are related to the same profession but to a different post or level of training or are actually the responsibility of a completely different professional group *(specify)* 5. Clarify if they were explicitly asked to carry out those functions by their superiors or supervisors? • *If yes, was it an oral instruction or wish or was it a written instruction? Where they offered any coaching or training to carry out these new functions or activities?* • *If no, why did they assume those functions or activities?* 6. Clarify if they derive any benefits and/or run any risks because of these extra functions? • *Specify what and from whom? Colleagues, patients, supervisors, councils …?* 7. Ask about the possible solutions for the current situation? |
| --- |
